# Teacher Scaffolding of Social and Intellectual Collaboration in Small Groups: A Comparative Case Study

**DOI:** 10.3389/fpsyg.2020.587058

**Published:** 2020-11-06

**Authors:** Elizabeth Kraatz, Manisha Nagpal, Tzu-Jung Lin, Ming-Yi Hsieh, Seung Yon Ha, Saetbyul Kim, Sangin Shin

**Affiliations:** ^1^Department of Educational Studies, The Ohio State University, Columbus, OH, United States; ^2^Institute of Education, National Chiao Tung University, Hsinchu, Taiwan

**Keywords:** collaborative discussion, relational equity, participatory equity, teacher scaffolding, idea building

## Abstract

This comparative case study features two small groups of students engaging in collaborative dialog about social issues. Based on social constructivist theories, the two groups were compared across three major components of the small groups system: social dynamics, intellectual collaboration, and teacher scaffolding. Our goal was to holistically analyze these small group processes to understand why some small groups were highly successful while others were not, even within the same intervention and with the same teacher. Successful groups were those in which all students were able to access the conversational floor, many ideas were considered, students were able to share ideas and discuss collaboratively, and students were able to raise multiple forms of social reasoning to support and explain ideas. Change in social reasoning essay scores prior to and after the intervention were also considered as evidence of group success. Results show that teacher scaffolding and existing student processes served to amplify one another reciprocally. The teacher heightened productive social norms when they were present, which then served to encourage productive intellectual collaboration. However, when productive group norms were not present, the teacher took increasing control over the group, which further hampered productive social and intellectual interactions.

## Introduction

Small group collaboration in classrooms is a complex and dynamic system in which various factors interact to influence student outcomes (Webb, 1982; Gillies, 2003). While many small group studies have overarchingly demonstrated the effectiveness of small group collaboration on students’ cognitive development (Foorman and Torgesen, 2001; Gillies, 2004; Webb, 2009; Webb et al., 2019), others have documented the heterogeneity in small group processes among students within classrooms or even under the same intervention practices (e.g., Webb, 1982; Barron, 2003; Webb et al., 2006; Volet et al., 2009). Much remains to be understood about why some small groups struggle more than others in small-group collaboration, specifically regarding how teachers orchestrate the dynamic and heterogeneous small group processes in the classroom (e.g., Jadallah et al., 2011).

There is also a lack of research that holistically considers small group collaboration processes. While quantitative methodologies have been valuable in identifying specific factors and their functioning, they are often limited in explaining how various factors interact with each other to constitute the dynamic system as a whole (Yin, 1994). As such, we employed comparative case study to analyze how two groups of students interacted with peers and their teacher throughout an established small-group intervention approach called Collaborative Social Reasoning (Lin et al., under review). This methodology enabled us to answer theory-informed questions while allowing us to address additional questions as they arose from observations (Yin, 1994; Merriam and Tisdell, 2016).

**FIGURE 1:**
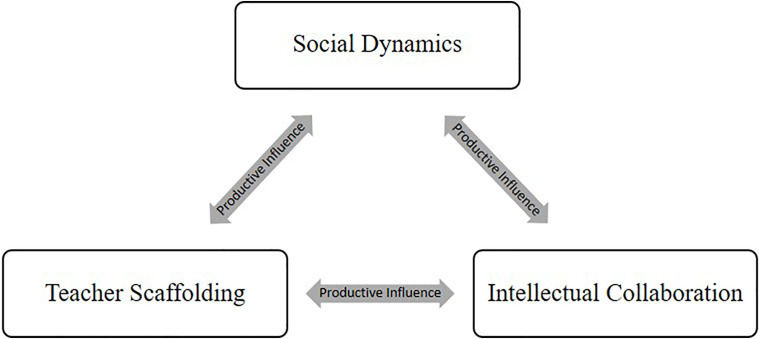
Dynamic system of small group collaboration.

Our aim was to understand why some small groups of students are highly successful in a collaborative small-group discussion intervention while others are not, even when groups seem comparable and students were taught by the same teacher. Successful groups showed strong collaboration, reasoning, and social interaction. We primarily focus on the processes of teacher scaffolding, social dynamics of the groups, and level of intellectual collaboration during collaborative small-group discussions and how these factors interact and vary between collaborative small groups (Figure 1).

While considerable literature exists in each of these areas, there is little that examines all three within a holistic system, making the contribution of this paper unique. Although we cannot assume that these processes would be generalized to outside of the systems we are studying, our findings can point to critical processes by which teachers facilitate small group collaboration, which can inform effective instructional practices in the future.

## Literature Review

### Small Group Discussion as a Dynamic Social System

As shown in Figure 1, we conceptualized small group collaboration in the classroom as a dynamic social system constituting processes of teacher scaffolding, social dynamics, and intellectual collaboration. In this model, social dynamics, such as turn-taking and ability to access the conversational floor, influence intellectual collaboration by ensuring that all ideas are heard and considered. Intellectual collaboration, or the extent to which students build knowledge upon the ideas of others’, influences social dynamics by providing a collaborative, constructive conversational floor for peer relations to grow and social skills to develop. The teacher’s role is to scaffold both of these processes, but these processes can also influence the ways in which the teacher provides scaffolding to the groups. Based on social constructivist theories (Vygotsky, 1934), this model conceptualizes learning as engrained in the social environment in which the learning happened (Adams, 2006); in this context, a small group of collaborating students within a classroom that is within a society. The model is based on prior work reviewed in the next sections and was used to structure our in-depth analysis of the two groups’ collaboration.

#### Social Dynamics

The ways by which students interact with one another and social relationships with peers can impact students’ academic development (Wentzel and Watkins, 2002; Buchs et al., 2009; Lee and Shute, 2010). For example, being accepted by peers can motivate students to engage in learning activities and display socially appropriate forms of behavior in group learning (Wentzel and Watkins, 2002). These studies emphasize how social and academic processes coalesce to influence academic outcomes including engagement and problem solving. Vygotsky (1934) also argued about how cognitive development occurs when individuals are tasked with a problem or activity that can be accomplished through concept formation with others. Furthermore, social discourse around the concept enables students to enhance and refine their conceptualization further than they would individually be capable of.

However, the goal structure of the social activity is important. Roseth et al. (2008) showed that collaborative design can be beneficial for students’ peer relationships. They found that cooperative goal structures, when individuals’ goals are inextricably linked and reliant on peers’ goals, promoted positive peer relationships more than either competitive or individualistic goal structures. These positive peer relationships, then, would further enhance the productivity of the group via enhanced social learning. As a result of these findings, we expected the social and cognitive processes within small group discussion to interact to produce greater learning than can be explained by either factor alone, presuming that both exist and are productive in nature. This is represented by the social dynamics and intellectual collaboration boxes in Figure 1.

For collaborative learning to occur effectively, the teacher must create equal opportunities for everyone to engage in constructive discourse, and students must take responsibility for advancing the group’s understanding by building on each other’s ideas/thinking (Hmelo-Silver and Barrows, 2008). Teacher scaffolding, then, is any teacher move that promotes students’ building and awareness of conceptual understanding (Boyd and Markarian, 2011). Effective construction of knowledge involves group effort that requires an intricate balance of turn taking, meaning making, and reflection. However, there is research that shows that it is not easy to maintain this balance and that discussions may move quickly from equitable to inequitable (Esmonde, 2009; Engle et al., 2014; Shah and Lewis, 2019).

In their empirical study, Shah and Lewis (2019) emphasize that equity in collaborative learning must be maintained in two ways: relational (the extent to which students demonstrate respect for their peers) and participatory (fair distribution of participation opportunities and participation itself in collaborative learning). Their research suggests that equity cannot be conceptualized as binary. This suggests that a collaborative process cannot be statically inequitable or equitable but is constantly in a state of flux and contingent on various factors, such as nature of the task, participation structure, relative content knowledge between collaborating students, students’ uptake, and teachers’ abilities to moderate these collaborations. Another interesting finding brought out by Shah and Lewis’s (2019) analysis of social interactions in collaboration shows that the net effect on equity of a single interaction is usually very small and it takes a series of small moves which can eventually amplify inequity over time and negatively influence the collaboration. This is important when analyzing factors that lead to either the success or failure of certain groups; inequity is less likely to appear as obvious statements of disrespect or disregard. Instead, it is more likely to present as many small, imperceptible interactions that accumulate over time.

Boaler (2008) further explored the idea of relational equity by describing three areas in which relational equity is perceptible in classrooms: respect for people’s ideas, leading to positive intellectual relations; commitment to the learning of others; and learned methods of communication and support. Boaler’s (2008) study was conducted to explore major differences in achievement, behavior, and culture between three urban high schools with similar populations. They wanted to explore why one school’s incoming freshmen began with the worst math test scores in the district but graduated with the highest. Their study found that high relational equity was the main difference between these schools and contributed substantially to the students’ conceptual learning. These students were devoted to effective, equitable communication to ensure that all collaborators thoroughly learned the material. As a result, students saw learning gains beyond those of otherwise comparable peers at other area schools (Boaler, 2008).

Participatory equity refers to students’ access to the conversational floor. Engle et al. (2014) defined participatory equity as “the degree to which the participant can initiate turns when desired, complete them without interruption, and control who else has access to the floor” (p. 8). The conversational floor, then, is “an evolving, socially negotiated space in which one or more particular people is allowed to present conversational contributions” (Engle et al., 2014, p. 253). In the context of small group discussion, participatory equity is achieved when group members have equal access to the conversational floor at will and without interruption.

Considering equity more broadly, Esmonde (2009) analyzed collaborative group work in mathematics classrooms and found that “expert” students tended to dominate certain collaborative activities. Engle et al. (2014) went further and proposed other factors that influence level of control and participation in collaborative discussions. They proposed a theoretical framework with five components to explain why some students tended to have greater influence in group discussions over others. Their findings suggest the following factors influence the level of participation, turn taking, and uptake of students’ ideas: (1) the negotiated merit of each participant’s contributions (i.e., the merit of student’s ideas is negotiated among group members rather than through any objective criteria); each participant’s (2) level of intellectual authority, (3) access to the conversational floor, (4) level of spatial privilege (physical placement, body language, etc.) and (5) level of influence in the discussion. They strongly recommended teachers and researchers consider all these factors when evaluating collaborative discussions or designing classroom activities. Overall, ensuring effective collaboration and uptake is not straightforward, and both teachers and students play important roles in balancing these discussions. The teacher can encourage provision of equal opportunities for students and facilitate connections between students’ ideas, whereas students need to focus on building knowledge and interacting productively with one another.

Relatively few studies have explored the influence of collaborative discussions on peer interactions and social experiences or the opposite (i.e., the influence of peer interactions on collaborative discussions). Anderson et al. (2001) found that when students participated in discussions with open participation, they tended to influence each other’s ways of thinking and phrasing arguments more than when the discussions were teacher-controlled. Lin et al. (under review) found positive impacts of collaborative discussions on classroom relationships, but casual mechanisms and influencing factors have yet to be explored.

Overall, collaborative discussions have been shown to provide students with opportunities to learn from one another, experience varied methods of communicating, make sense of social experiences, and remain engaged and motivated (Laal and Ghodsi, 2012; Wu et al., 2013). As mentioned above, social norms, group dynamics, and equity can all impact the effectiveness of group functioning. This study will explore if and how peer interactions and group dynamics influence the quality of collaborative small-group discussions in conjunction with cognitive processes. Next, the intellectual subsystem is considered.

#### Intellectual Collaboration

Idea building, also referred to as knowledge building, refers to collaborative efforts to construct, transform, and refine collective knowledge through discourse (Hmelo-Silver and Barrows, 2008, pp. 48–49). This definition encompasses an infinite range of situations in which discussion helps students build conceptual understanding. However, while a great deal of work has been done on idea building in various collaborative learning settings, much of it has focused on student interactions centering around one “best” or “correct” answer (e.g., Webb, 2000; Sfard and Kieran, 2001; Hmelo-Silver and Barrows, 2008; Ing et al., 2015). The assumption underlying this body of work, much of which has been conducted in math classrooms, is that students should arrive at similar understanding around a common answer, which places students in a helper/helpee or expert/novice relationship in which some students are more, and others less, knowledgeable. This is problematic as, in many dilemmas, equally valid reasoning may result in several equally valid conclusions. This is not to say that all ideas should be assumed equally valid, but rather that there may be no “right” answer but instead be multiple well-justified, well-reasoned conclusions or courses of action based on different values, ideas, or perspectives. In these cases, it is more important that students are able to consider, critique, refine, and respond to ideas around a certain topic, rather than that they are able to clearly and effectively explain their solution to a problem. For this reason, we consider idea building as an index of intellectual collaboration, which we define in this study as a process that includes questioning an idea, proposing a new idea, responding/adding to an existing idea, or raising evidence for or against a proposed idea. This process may take multiple forms, and students are not working toward a particular answer, but are rather working to weigh different perspectives, ideas, and domains of knowledge provided by others and then formulate those into their own conclusion (Cazden, 1988; Killen, 2007; Hitti et al., 2014; Chiasson et al., 2017).

Two processes of idea building/intellectual collaboration have been found to be related to students’ learning: provision of detailed explanations, and engagement with others’ ideas (Webb et al., 2014; Ing et al., 2015). Vygotsky’s (1934) theory emphasizes the importance of group members being willing to listen to each other’s ideas and respecting it, in order to support idea building. These positive dynamics would help shape one’s own ideas and connect it to others. Warner (2008) suggested that students build knowledge in several ways: explaining, reorganizing, or building on an idea; questioning to show that an idea is valid or invalid; connecting or proposing multiple representations of the idea; applying the same idea in multiple contexts; and raising hypotheticals.

#### Teacher Scaffolding

Wood et al. (1976) were among the first to apply the term “scaffolding” to education when they explained how adults used varying strategies to help young learners with problem solving. Examples of cognitive scaffolding include slowly increasing the complexity of the problem at hand, encouraging higher level thinking, directing students’ attention to critical features, or modeling reasoning or problem solving. Social scaffolding includes managing group dynamics by helping students support one another, ensuring equal contribution/participation by all, and helping students stay on task and maintain direction toward the goal (Belland et al., 2013). Regardless of the focus of these strategies, they consistently encourage students to build awareness of and depth in their conceptual understanding of the topic at hand (Boyd and Markarian, 2011). In the context of classroom discussions, research has identified principled strategies of cognitive scaffolding to enhance the quality of discussion (Chinn et al., 2000; Alexander, 2017; Howe et al., 2019). For example, Webb (2009) found that probing students’ explanations to uncover details of their thinking and problem-solving strategies is an effective scaffolding strategy to promote learning. To date, however, there is comparatively less research on the non-academic scaffolding of dialogic discussion or how teachers can support discussions in ways that are beyond prompting for reasoning alone (Puntambekar and Kolodner, 2005; Belland et al., 2008; Belland et al., 2013).

The effectiveness of scaffolding is dependent on various factors such as contingency (appropriateness of support based on student needs/ability), context, timing of fading, and nature of the task (Howe, 2013; van de Pol et al., 2015, 2019). van de Pol et al. (2015) showed that a combination of the above-mentioned factors together influences student outcomes. Their study highlighted that contingency alone does not ensure effectiveness and that the frequency with which the teacher provides support and the nature of the task is also important in determining the effectiveness of teacher scaffolding. van de Pol et al. (2019) used qualitative analyses to further show that students’ uptake of contingent support was sometimes hampered by untimely fading of the support and that it was most effective when the support faded gradually. In another study, Howe (2013) showed how the efficacy of teacher scaffolding is influenced by the nature of the task. For instance, in group work that requires abstraction and resolving different opinions, teachers are encouraged to use probing to encourage students to explain their reasoning while providing support as students move toward resolution. Overall, these studies highlighted that teacher scaffolding is influenced by many factors and that the nature of scaffolding can vary depending on the task and classroom context.

Teacher’s scaffolding not only influences group processes but can be shaped by group processes, indicating a bidirectional relationship. This means that the teacher both influences and is influenced by the students she/he is scaffolding. Webb et al. (2006) found that students largely mirrored teachers’ modeled discourse and communication patterns. While such research showed how teacher scaffolding can influence student outcomes, Chen and Jiang (2004) demonstrated how the opposite can also play a role, that is, how student group dynamics influenced the way the teacher provides scaffolding. In their study, Group A had better group dynamics and coordination, thereby allowing the teacher to play the role of a ‘follower’ and focus on providing cognitive structuring. Group B, on the other hand, lacked effective communication and coordination, so the mentor had to play the role of an initiator while increasing focus on social psychological aspects such as sensitivity, encouragement, and humor, rather than focusing on cognitive elements. This highlighted the reciprocal influence between teacher scaffolding and student group processes.

Teacher scaffolding is complex and multifaceted, as teachers both influence and are influenced by social processes in the classroom. While many factors may influence the effectiveness of scaffolding, it has been consistently shown that what is most critical is the level of conceptual consideration the teacher is helping the students interact with. This facilitation might take different forms within different contexts. Based on the literature above, we define ideal scaffolding as the timely use of teacher strategies to temporarily support students’ cognitive needs and social needs in a small group discussion (including probing, modeling, direction maintenance, supporting autonomy, frustration control, monitoring group dynamics, etc.) until students gain sufficient skill to engage in a productive discussion (Webb, 2009; Belland et al., 2013).

### Collaborative Social Reasoning: A Collaborative, Small-Group Intervention

This case study is situated within a dialogic, social reasoning intervention called Collaborative Social Reasoning (CSR). This approach is informed by the substantial literature on Collaborative Reasoning (Chinn et al., 2001; Reznitskaya et al., 2009). The fundamental assumption underlying the approach is that knowledge is socially constructed through meaningful and authentic interactions (Vygotsky, 1934). The context of CSR was selected for several reasons: It has been shown to be effective at improving students’ social reasoning (Lin et al., under review); it allows for in-depth analysis of students and teachers engaging in collaborative, democratic discussions; and it is based on ambiguous social-moral dilemmas for which there is no single or simple answer. This enables students and teachers to engage in reasoned argumentation about social issues in genuine, democratic ways, thereby providing ample opportunity for qualitative analysis of the discussions (Lin et al., under review).

Centered specifically on complex social-moral issues, CSR adopts four theoretical and research-driven design principles aiming at creating critical dialog with purposeful and meaningful collaboration (Lin et al., under review): (1) collaborative argumentation, (2) positive social norms, or baseline expectations of respectful and productive interaction, (3) teacher facilitation, and (4) multi-faceted literary texts (Lin et al., under review). The first, collaborative argumentation (Chinn and Clark, 2013), focuses on the goal of building understanding with each other rather than convincing others of a particular viewpoint. Positive social norms included turn-taking, respect for all ideas and opinions, sharing of the conversational floor equitably (Engle et al., 2014), and open participation that enable students to share ideas freely without worrying about teacher evaluation (Au and Mason, 1981).

The purpose of teacher facilitation is to ensure that (1) all students in the group comprehend discussion texts and questions, (2) discussions do not remain at surface levels (e.g., checking facts) but involve higher-level thinking (e.g., critical thinking, metacognition) and (3) group dynamics are effective at supporting high-level cognition and collaboration. In addition, teachers play an important role as a facilitator, who gradually fade their facilitation as students become more independent thinkers.

The use of multi-faceted text is the final design principle of CSR. Fictional stories were selected, excerpted, and adapted in order to facilitate collaborative argumentation with peers (Walton, 1998); stimulate social perspective taking (Bakhtin, 1981); and help students connect thought and action. To achieve these ends, storylines were linked to current social or political issues in the students’ everyday life (e.g., fitting into a social group at school, experiences of racism). The stories were designed to provoke students’ knowledge and experiences about social issues in order to promote collaborative and equitable discussions.

### Research Questions

This study applies a case study approach, in which in-depth analysis is completed to holistically explore small group processes as a dynamic social system, to uncover the ways in which teachers carry out these responsibilities in collaborative small group discussions, while also relating these practices to student learning. The present study aims to move beyond the existing work by examining not only the scaffolding that exists, but also how teacher scaffolding interacts with the group dynamics and intellectual collaboration within a discussion-based small-group intervention.

The present study aims to explore the interactions between cognitive, social, and scaffolding processes within small group discussions. This will enable a much fuller understanding of how teachers serve as holistic facilitators in the discussion, rather than simply as enhancers of cognition. Our aim is to explore the following research questions:

• What are the major differences in patterns of social dynamics and intellectual collaboration throughout the course of six CSR discussions between a high-performing (demonstrates high-level, collaborative dialogic, and productive social dynamics) and a low-performing group (demonstrates lack of high-level, collaborative dialogic and productive social dynamics)?
• How are the patterns of social dynamics and intellectual collaboration related to the teacher’s scaffolding strategies?

## Materials and Methods

### Source of Data

The data were drawn from a larger project in two urban, Midwestern public schools in the United States. The purpose of the larger project was to develop a small-group discussion approach called Collaborative Social Reasoning (CSR), and to examine its impacts on students’ interpersonal competencies and social reasoning. Participating teachers engaged in a 2-day workshop to learn about CSR principles and strategies. Scaffolding strategies were suggested to the teachers, and they were encouraged to give students control of the discussion as much as possible. As a result, teachers were exposed to similar scaffolding strategies but were allowed to implement them in different ways and to different extents.

The larger project contained six fifth-grade classrooms in the treatment condition and six classrooms in two control-comparison conditions. Four small groups were formed in each of the six treatment classrooms, totaling 24 small groups and a total of 144 discussions. The research team transcribed discussions two, four, and six from all 24 small groups (mean age = 10.94 years, *SD* = 0.41). As a result, analyses that required transcripts were completed based on these weeks’ discussions. However, all videos associated with the study cases were analyzed in depth to uncover differences in small-group discussion processes between the cases. All students were assigned a pseudonym, which are used through the remainder of the paper. Students and teacher were told about the purpose of the larger project: to understand how CSR works and affects students’ learning in an authentic classroom setting. Pseudonyms were also used to represent student and teacher identities in conversations and correspondence about the project. The data were also stored using pseudonyms and/or student ID numbers.

#### Case Selection

For the purpose of this comparative case study, we selected one high-performing and one low-performing group based on the following procedure and criteria. First, two expert researchers independently reviewed videos of the final (week 6) discussion for all 24 small groups and nominated those that were particularly productive or struggling. Criteria evaluated were number of perspectives considered, nature of social interactions, and depth of social-moral reasoning. Groups that considered many perspectives, had positive social interactions, and showed great depth in their reasoning were nominated as high achieving. Groups in which this was most notably absent were nominated as low performing. There was more than 75% overlap in the groups noted by the two researchers. Groups that were nominated by both researchers were presented to the research team via video clips of the week 6 discussion. The research team was shown the clips without indication of prior evaluation and asked to rate the group’s success in the discussion. Of the groups unanimously agreed to be high- or low-performing, two of the most contrasting groups came from the same teacher in the same school. These two small groups were chosen for the study because they were unanimously agreed to be high- or low-performing and they allowed us to examine teacher’s roles under the same school and cultural contexts, reducing extraneous influencing factors.

For the intervention, groups were designed to be heterogeneous to best represent the classroom composition. We used pretest data collected from the larger project to identify shy, aggressive, popular, and rejected students. This information, along with students’ academic level, race, and gender, were used to create heterogeneous groups within each classroom. For more information on these scales and the group creation procedure, please see Lin et al. (under review) and Nagpal et al. (2020). In the struggling group, there were two females and four males. Both females and one male were White and the other three students were Black. In the high-performing group, there were three males and four females. Two males were Hispanic and the other was Black. All the females were White. The teacher was a White female in her first year of teaching.

To establish that the two groups of students were comparable at the outset of the intervention, we compared three of the major pre-test measures drawn from the larger project: (1) peer acceptance, defined as the extent to which peers like to work and play with each student, was assessed using a peer nomination approach in which students rated each of their classmates according to how much they liked to play or work with that peer on a scale of 1 (not at all) to 10 (very much) (Parker and Asher, 1993); (2) social reasoning, defined as knowledge about the complex social world (Turiel, 1983), was assessed by an individual essay task, which was coded based on a coding scheme designed to examine the number of perspectives students considered in the essay (see Kraatz et al., 2019 for more details about the coding scheme; inter-rater reliability α = 0.88); (3) academic achievement, which was based on students’ 4th grade state standardized language arts scores. Independent samples *t*-tests were conducted to compare the groups’ average peer acceptance, social reasoning, and academic performance. There were no significant differences in pre-test peer acceptance [*M* = 4.46, *SD* = 1.18; *t*(10) = −1.46, *p* = 0.18], social reasoning [*M* = 0.82, *SD* = 1.33; *t*(9) = 0.33, *p* = 0.58], or 4th grade standardized test score [*M* = 693, *SD* = 28.67; *t*(9) = 0.59, *p* = 0.57]. Over the course of the intervention, the high-performing group significantly increased their social reasoning score [Time 1 *M* = 0.86, *SD* = 1.57; Time 2 *M* = 2.71, *SD* = 1.25; *t*(6) = −2.64, *p* = 0.04], while the low-performing group did not [Time 1 *M* = 0.08, *SD* = 0.96; Time 2 *M* = 0.50, *SD* = 0.58; *t*(3) = 1, *p* = 0.39]. The average length of discussions in both groups was 24 min, indicating similar time spent in the small groups over the 6 weeks.

### CSR Procedure

The CSR intervention occurs over 6 weeks, and students read and discuss one story related to social exclusion each week. Each discussion focuses around a “big question,” which features an ambiguous social moral dilemma. A researcher was present in each classroom during all CSR discussions to monitor the fidelity of the implementation. Prior to the intervention, a norm-setting session lasting about an hour was conducted by a researcher and the teacher within each classroom to elaborate expectations for critical, collaborative, and respectful dialog and give students a chance to set norms for their own discussions. Teachers were trained to facilitate and encourage CSR norms while scaffolding students’ argumentation. However, it was emphasized that the discussion belonged to the students; they controlled the ideas, flow, and turn-taking. Furthermore, in order to promote equity, teachers were encouraged to help students problematize content by encouraging questioning, challenging, and other intellectual contributions; share authority by making students genuine participants in classroom discourse; ensure accountability to others’ and intellectual norms; and provide access to needed resources (Cornelius and Herrenkohl, 2004). Students were encouraged to consider all possible viewpoints before arriving at their own conclusion, with no need for group consensus. The discussion then ended with a teacher-led debriefing session in which students reflected on their individual and group performance with respect to their goals. The group then discussed possible goals for their next discussion.

### Group Comparison Approaches

Once the groups were selected, the first and second authors engaged in in-depth analysis of the six discussion videos for each group. Following Creswell and Poth’s (2018) data analysis procedure, we first took detailed notes and completed memoing of the data. Weekly meetings were held in order to compare notes and consolidate areas of interest. We then examined the notes, memos, and codes from the twelve total discussions and compared these to the three major themes previously identified in the literature. Within each of the three major categories, we used pattern matching to examine the ways in which the teacher interacts with both groups of students and then compared the similarities and differences in these interactions (Yin, 1994). We found several areas of difference within the theoretically defined categories: within social dynamics, we found differences in social equity, which can further be broken into participatory and relational equity. Idea building and resulting collaborative arguments differed within the “intellectual collaboration” umbrella, and major differences in teacher scaffolding were noted for both social and cognitive scaffolding moves. The results of the memoing were used to conduct more detailed literature review to guide our in-depth analysis. The qualitative and quantitative analyses were used together in order to triangulate findings and increase validity (Atkins and Wallace, 2015; Merriam and Tisdell, 2016).

#### Social Dynamics

Two aspects of social dynamics were observed to differ between the high- and low-performing groups: relational equity and participatory equity (Shah and Lewis, 2019). Relational equity refers to respect for others’ differences, ideas, perspectives, and actions (Boaler, 2008). One researcher notated all instances in both groups in which students demonstrated consideration of others’ learning, ideas, and perspectives. These occurrences could be explicit statements such as “oh, I never thought of it like that!” or more subtle, seen through engagement in intellectual conversation in which students considered the ideas of others in relation to their own ideas, demonstrating distribution of power within the group (Cornelius and Herrenkohl, 2004). Each transcript was examined at the turn-of-talk level–each student turn of talk was examined and if it included that student showing relational equity, it was coded as such. One researcher coded all the transcripts, and another researcher independently reviewed the entire coding for reliability. There was 90% agreement between the researchers and any disagreements were discussed until 100% agreement was reached.

In terms of participatory equity, we examined the extent to which students accessed, or were unable to access, the conversational floor (Engle et al., 2014) across the six discussions. This was completed by coding all interruptions that occurred within each discussion. Instances in which students uttered exclamations or other phrases that did not disrupt the flow of the conversation (interjections) were not counted because there was not a genuine conflict for the conversational floor. Examples of interjections include simple agreement (e.g., ‘yeah,’ ‘uh-hm’) or other short, non-essential turns (e.g., ‘That’s weird’). Each interruption was coded during video analysis; videos were initially coded in order to examine the flow of the conversation, which is difficult to do from a transcript. Approximately 25% of all interruption codes were verified by another researcher for accuracy. There was 100% agreement between the two researchers.

Each time two or more students entered the conversation in a way that created a conflict for the floor, an interruption was coded. This could be one student interrupting another, two students initiating a turn of talk at the same time, or students talking over one another. We coded disruptions to the conversation with the assumption that, unless the conversation is disrupted, students are able to gain the floor when they choose to. We did not assume that all students desire to speak with the same frequency, so we did not consider the number of turns each student takes as a measure of equity. During the video analysis, there was no evidence that students wanted but were unable to gain the floor except where interruptions occurred (no students showed signs of wanting but being unable to speak), so this represents a reasonable estimation of equitable access to the floor. Each interruption was further coded as amicable or competitive.

Amicable conflicts occurred in two forms. First, two students may begin speaking simultaneously and one then cedes the floor to the other. This indicates that the students were aware of peers’ speech and saw value in releasing the floor even though this meant their own idea would not be heard immediately. Second, amicable conflicts occurred when one student interrupted another, realized their interruption, and ceased speaking. This was often accompanied with a “sorry” or a nod to the person being interrupted. This shows students’ recognition of their peers’ speech and the equitable norms that require respectful turn-taking. All other interruptions that did not involve the teacher were coded as competitive and tended to take the form of one student interrupting another and both trying to be heard at the expense of the other. Sometimes, the original speaker abruptly ended their attempt to share rather than trying to compete for the floor. Looking at amicable conflicts in addition to total conflicts enables deeper examination of social dynamics; even highly-functioning conversations may have instances of simultaneous speech or accidental interruptions, especially if participants are eager to share, so it can be beneficial to separate these interruptions from those that restrict access to the floor for quieter group members.

Instances of student inviting or encouraging one another to share were also coded as participatory equity. This was done in tandem with the relational equity codes. All codes were completed by the first author and reviewed by the second. There was greater than 90% agreement between researchers. Discrepancies were discussed until 100% agreement was reached.

After coding was completed, videos were again reviewed and notated with observations and explanations that the codes alone could not encapsulate. We used explanation building methods to examine reasons for the findings from the coding. Explanation building methods are a procedure in which various possible explanations are considered iteratively to build an explanation within a case study (Yin, 1994). We completed this procedure to examine the possible impacts that various teacher behaviors had on the functioning of the small groups.

#### Intellectual Collaboration

To examine intellectual collaboration, we first considered how often students were building upon each other’s ideas, versus simply sharing without co-construction. In order to examine this, all student turns of talk were analyzed and those that questioned another’s idea in a constructive way, built upon an idea, or provided a different viewpoint or piece of evidence on an idea were counted. We call this code “idea-building.” Simple agreement or disagreement, as well as agreement or disagreement that simply stated an alternate idea without relating that idea to the previous were not counted because they demonstrated little intellectual collaboration. This coding was intended to show how students’ ideas related to one another’s. All codes were completed by the first researcher and reviewed by the second author to ensure consistency and validity. Initial agreement was approximately 90% and any disagreement was discussed until 100% agreement was reached.

Arguments, or claims made about the topics of conversation, made by individual group members were also analyzed. This was done by summarizing all arguments into tables by group member. In order to summarize the trends in the two groups, the number of ideas professed by each group member was counted. For this analysis, we did not conduct a quality evaluation of whether the idea professed was reasonable or made sense; instead, we were simply looking at how many ideas were put forth by each group member during the discussion. Then, the tables were examined to identify trends in reasoning in both groups. For instance, did students consider multiple possible viewpoints in the discussion or simply repeat arguments for one or two? Were they able to support their ideas with evidence? These trends were examined in depth for discussions two and six to compare the starting and ending points in the group’s intellectual collaboration. Week 1 was not included because we assumed students needed time to adjust to the novel discussion format. All points made in the discussions were summarized by one researcher and the first and second author analyzed them collaboratively.

#### Teacher Scaffolding

Teacher’s talk was analyzed through creation of tables which placed the teacher’s speech in each group side by side for comparison. Because teacher’s turns of talk were relatively few in each discussion, we were able to examine all teacher turns of talk in each group to identify similarities and differences. This made differences in teacher interactions with each group apparent and similarities and differences salient. Teacher’s turns of talk (excluding interjections and acknowledgments) were categorized by function. Nine different types of teacher cognitive scaffolding emerged: asking open questions, redirection to the Big Question, modeling reasoning, playing devil’s advocate, presenting hypotheticals, prompting individual students to speak, asking clarifying questions, and providing low-level support (e.g., vocabulary, giving instructions). All teacher turns of talk were analyzed collaboratively by the first and second authors, who discussed the key features and differences until 100% agreement was reached. The videos were then revisited in order to examine the ways in which students reacted to the teacher’s input. We particularly focused on the ways in which the teacher interacted with student ideas and how she built upon them or asked students to build upon her ideas.

To examine the role of the teacher’s social scaffolding within these small groups, we also coded each instance of the teacher granting the floor to a particular student (participatory equity) or engaging in promoting relational equity (promotion of value for and validity of varied viewpoints). Because the teacher holds a unique position in which she can prioritize the contributions of some students over others, participatory inequity was also coded, which represents instances in which the teacher puts the contributions of one student or her own ideas above those of another student. This coding was completed on the transcripts for discussions two, four, and six. All transcripts were coded by the first author and an independent researcher coded 33% percent to ensure reliability. Cronbach’s alphas were 0.85, 0.83, and 0.94 for relational equity, participatory equity, and participatory inequity, respectively. Examples of these codes can be found in Table 1. After coding, videos and transcripts were reviewed in order to identify the ways in which teacher equity moves function within the group. Areas of focus were the group peer dynamics and the interactions and dynamics of how the students shared ideas in relation to teacher talk.

**TABLE 1:** Teacher equity codes.

Relational equity examples (demonstrate value for and consideration of others’ perspectives)	Participatory equity examples (increase equitable access to the conversational floor)	Participatory inequity examples (decrease equitable access to the conversational floor)
“Oh, that’s a good thought!” Jordan: I change my answer to yes. Teacher: Okay, I’m just saying that some can say. I’m not saying that it’s the right answer. Right? [1] [1] [to Jordan] Why do you agree?	“What are you thinking over there, Cameron?” Jordan: I change my answer to yes. Teacher: Okay, I’m just saying that some can say. I’m not saying that it’s the right answer. Right? [1] [1] [to Jordan] Why do you agree?	Teacher: Why do you think that? Peyton: [Be]Cause emotional harm.// T: Use this is to help you answer the big question should she forgive her? (Floor released to Ali) Spencer: My viewpoint is starting// Teacher: I didn’t mean to cut you off I’m just trying to get you to think from the other perspective. Could you ignore that? Because the sixties were a rough time to be down south. They would kill you, beat you, that picture of the thing by the fire hydrant, blowing water at them. Elliot: Yeah it even said here in page seven that like that one of their um uncles and stuff said that they’re gonna-if you go down south they’re going to lynch you which means that they’re basically going to kill you or hang you. Spencer: Um// T: Tricky, isn’t it?

## Results

### Social Dynamics

The raw numbers of social dynamics codes are presented in Table 2. The low-performing group had a discussion with no positive social dynamics in week 4 and showed general decrease in all codes from week 2 to week 6. The high performing group, on the other hand, showed an opposite trend, with increases in all fields from week 2 to week 4 and again in most fields from week 4 to week 6. Discussion lengths are provided to give context to the raw scores. Since all discussions were not equal in length, it is probable that shorter discussions may have fewer codes. However, Table 2 indicates that length alone does not explain the differences between groups.

**TABLE 2:** Counts of student social dynamics codes.

	Low group			High group		
Week	2	4	6	2	4	6
Discussion length (minutes)	29	17	28	21	25	32
Relational equity (count)	3	0	2	3	6	16
Participatory equity (count)	11	0	6	1	4	1

The low-performing group generated more conflicts for the floor than the high-performing group over time (Table 3). Additionally, the proportion of amicable conflicts was lower in the low-performing than the high-performing group. This indicates that, in addition to fewer overall conflicts in the high-performing group, they were also able to attend to peer’s speech and adjust their own accordingly. It may seem that the low-performing group improved their interactions over time, as the number of conflicts for the floor peaks early in the intervention. However, this does not seem to be the case; instead, the conflicts were reduced as students became less participatory in the discussion. This was observed during the video analysis. Students in the low-performing group showed signs of low engagement including staring into space, increased fidgeting, or even putting their head on the table. The conversational floor was more open, but not because students were improving at sharing it.

**TABLE 3:** Conflicts for the floor.

	Low group			High group		
Week	2	4	6	2	4	6
Discussion length (minutes)	29	17	28	21	25	32
Total conflicts	60	16	15	6	8	6
Proportion of amicable conflicts	0.20	0.31	0	0.83	0.63	0.67

Further considering these conflicts for the floor, in the low-performing group conflicts tended not to be directly related to the content of talk; students were talking over one another in a competitive way (trying to have their own idea heard) rather than in a collaborative way (building on one another’s ideas). As shown in the most conflict-dense segments of each group’s week 4 discussion in the Appendix, there was a lack of relational equity in the low-performing group, as students were prioritizing their own ideas at the expense of their classmates’ and were not demonstrating respect for the contributions of their peers. Furthermore, researchers’ memos of video analysis documented that in the low-performing group there was, at times, clear animosity between group members, in facial expressions (making a face when someone talked) or body language (turning away from a group member to exclude them from the discussion).

The social dynamics in this group were not always negative; students in this group did encourage one another to speak, ask one another questions, and intentionally attempt to include those group members that participated less frequently, as can be seen in the conflicts for the floor data above. However, these positive social interactions decreased over time, and individual students seemed more and more frustrated with the discussion process.

In the high-performing group, the students contributed more equitably. There were still students who participated more often than others, but the disparity was less severe, and the teacher did not seem to feel it necessary to intervene in participation. In the early discussions, two students served as leaders, showing imbalance of intellectual authority (Engle et al., 2014). However, this is not apparent in the later discussions, with the majority of ideas being addressed to the group as whole and no discernible differences in intellectual authority. In this group, when conflicts for the floor occurred, the students seemed aware and apologized for interruptions or yielded the floor to a peer. There is clear respect for the input and ideas of others, without apparent imbalance of power, showing high levels of relational equity. An example of the respectful exchanges that were the norm in this group is below. In this excerpt from the high-performing group’s week 2 discussion, we see an example of an amicable conflict, in which one student, Cameron, interrupts another, Spencer. Cameron then realizes the interruption, yields the floor back to Spencer, and waits until Spencer finished speaking to share her own point. This awareness of peer’s access to the floor seemed to increase collaboration within the group and promote increasing equity.

**Table:** 

**Spencer:**	Oh. But like- they’re two different teams and like most likely they’ll end up on the field together and that’s why it happened. I think that- [1] [1] Um, I think that um, Aki- I think that they could have prevented it from happening if like- if- I forget the girl that like she wasn’t going to tell on Shirley for being racist. [looks in text] Um I think her name is… I forget her name, but um she could have told the coach instead of making their team look better, she could have told the coach of what Shirley has been doing. To make sure it didn’t happen.
**Cameron:**	[1] I… go ahead. [1]
**Cameron:**	I think that Aki’s friends could have prevented this by not to happen because like they knew that Shirley was running in the baseline and then they would know that Aki could have gotten her and said like “watch out” and yeah.

Consistent with the result of participatory equity coding, we observed from the discussion videos that even though some students spoke less often than others, they were easily able to gain the floor when they chose to participate, and their body language indicated engagement in the conversation. Furthermore, most conflicts for the floor occurred in the midst of collaboration and are in the pursuit of idea building. Students in this group did not show visible signs of frustration with group dynamics and seemed to consider their collaboration as a source of pride, as seen by comments in their debriefing sessions. The excerpt below was taken from a debriefing after week six’s discussion, which shows that students reported experiencing growth in their own abilities.

**Table:** 

**Jaymie:**	We started to argue more.
**Spencer:**	Yeah, how we have our different opinions, and our different sides of the story.
**Harper:**	We went back in the text and looked for things that we could use to try to say.
**Teacher:**	Okay, so this question goes along with what we are talking about. So, remember, at the beginning of this group, we made class goals for all of us in the class? Which do you think we’ve improved the most? Like, you’ve seen the most growth? In which of those goals up there?
**Harper:**	Arguing more. Everyone participates. We used the text to support our answers, to support our opinion.
**Cameron:**	And then, we also that we explain our ideas clearly, and we didn’t mumble what we have to say.
**Elliot:**	We listen to both sides.
**Spencer:**	Yeah, we stayed on task.
**Cameron:**	And we respected each other.
	……..
**Spencer:**	I learned to give lots of details, and lots of reasons on my opinion, and my point of view on the story.
**Harper:**	I learned to respect what everyone had to say about their opinion.

### Intellectual Collaboration

In considering the idea building within the two groups, we observed a decrease over time in the low-performing group and an increase in the high-performing group. We observed students in the high-performing group increasing the collaborative nature of their contributions. The opposite happens in the low group. This can be seen in Table 4.

**TABLE 4:** Idea building.

	Low group			High group		
Week	2	4	6	2	4	6
Disc. length (minutes)	29	17	28	21	25	32
Idea building (count)	40	0	8	13	32	37
Idea building per minute	1.38	0	0.29	0.62	1.28	1.16

The number of arguments generated by each group member are presented in Table 5. One high group student was omitted from the table because they were absent in both week 2 and week 6.

**TABLE 5:** Number of arguments by group member.

Group	Student	Week 2	Week 6
Low	Jordan	23	20
	Ryan	14	16
	Taylor	2	3
	Peyton	30	11
	Ali	33	Absent
High	Cameron	7	14
	Elliot	4	36
	Harper	6	28
	Jaymie	4	29
	Parker	7	20
	Spencer	5	Absent

### Discourse Data Linking Social Dynamics and Intellectual Collaboration

In this section, we present qualitative evidence from CSR discussions demonstrating how the high- and low-performing groups changed in their social dynamics and intellectual collaboration over time. In the earlier discussions, students in the low-performing group held different initial positions to the Big Question and were able to voice their opinion and explain why they held it. This can be seen in the following excerpt from week 2, in which students are expressing reasons for their differing opinions on whether one character (Aki) should forgive another character (Shirley). Shirley hurt Aki because Aki is of Japanese heritage and Shirley’s father was killed by Japanese soldiers at Pearl Harbor. These students from the low-performing group discuss the characters’ emotion and its connection to the experiences of each, their own ideas about right and wrong, the role of “difference” in social interactions, characters’ rights, and how these factors were situated within the social-historical context. We do see an amicable conflict in this excerpt, as we see Ali yielding the floor to Jordan. However, this did not become the norm in this group, as is evident in Table 3’s data on conflicts for the floor.

**Table:** 

**Jordan:**	Shirley probably was just upset that her dad died, and… And she just was out of control, and she just hit the softball toward Aki.
**Ali:**	I disagree with you, because I think that Shirley shouldn’t have taken it out on someone else. I know she was probably upset, but it’s still not right to take her anger out on someone else.
**Ryan:**	Just because they’re different, doesn’t mean… She wasn’t the cause of what all happened. She didn’t plan for all of it to happen, and it’s not her fault she was born Japanese, and just because she’s that type of person… A Japanese person, doesn’t mean that she really has the right to hurt her.
**Peyton:**	Yeah, yeah, besides, war ended already. So I understand her dad died, but she needs to… I think… He’s passed away. You need to get over it.
**Taylor:**	Maybe she’s just trying to avenge her father.
**Peyton:**	Yeah, I know, but the Japanese and Americans signed a peace treaty. So why is there a reason that Shirley hit Aki? Why is there a reason that she hit her? [1] [1] I know her dad died, but everybody passes away sometime, and she needs to get over it.
**Jordan:**	[1] I think the reason that- [1]
**Ali:**	I- [to Jordan] You can go.
**Jordan:**	I think that she just was too just frustrated that her dad died, and she only had her mom, and she just didn’t plan to hit Aki, but she just was thinking that. [1] [1] That she was just thinking of her dad, and she just got out of control, and she hit Aki, and…

The high-performing group, on the other hand, started off with less intellectual collaboration between the group members. There were frequent pauses and students were not able to generate ideas as fluently as in the low-performing group, as seen in the week 2 excerpt below. These students discuss the idea of blame, characters’ desires and emotions, and story occurrences. However, the nuance, integration of ideas, and constructive flow that was present in the previous excerpt is not apparent here.

**Table:** 

**Parker:**	Well I think that um, Aki should not forgive Shirley because it’s not really her fault what happened, and she basically like blamed her for everything and it’s not her fault. She didn’t do anything.
**Spencer:**	Well yeah I understand but I kind of disagree with your answer because um that Shirley like she may have not well like they’re- what- I forget what grade they’re in…
**Cameron:**	Sixth.
**Harper:**	They’re probably just//
**Spencer:**	So like very young. Well they like- they may not have known what they were doing and why- so yeah.
**Jaymie:**	I agree with Parker because Shirley keep on like being mean to Aki.
**Cameron:**	I think that Shir- that Aki shouldn’t forgive Shirley because like if you like hurt somebody like Shirley did, then you probably don’t want to forgive them after they hurt you.
**Harper:**	Umm… I think that Aki should forgive Shirley um because they’re probably just both angry at each other and they probably just want to um… just get all of their anger out or something.
**Spencer:**	Well Aki isn’t like mad at Shirley she just like- she really don’t care about it. Because I bet that probably happened to her like multiple times.

However, over time, we observed less intellectual collaboration in the low-performing group. The majority of different perspectives were raised by two students, Jordan and Ryan, and, as the teacher focused her attention on the other group members, these ideas were often ignored. This led to little change in the contributions from Ryan and Jordan over time and less intellectual collaboration present in the group overall. Ryan and Jordan’s ideas were not picked up by others, who tended to focus on their own opinions. This is seen in particular in Jordan and Peyton’s comments in the following excerpt from week 6. The story for this week focused on a character (Dovey) whose brother (Amos) accidentally killed someone (Parnell) in preventing Parnell from further hurting Dovey, who was unconscious. The question is regarding whether Dovey should tell what she knows or allow the blame to be placed on another deceased character in order to protect her brother. Peyton is discussing the unsavory nature of Parnell, while Jordan is commenting on the unfairness in the story. However, these students are not able to connect their parallel ideas into a coherent overarching conclusion.

**Table:** 

**Jordan:**	If you keep it a secret, then the dude that was there when he has to go to jail for no reason, [1] [1] when he didn’t do it. And they think that he killed Parnell,// but he didn’t.
**Ryan:**	[1] Well… [1]
**Peyton:**	//Hey Jordan, Hey Jordan. Um… well two dogs couldn’t do it because they wouldn’t be able to lift something that… they wouldn’t be able to lift that?
**Jordan:**	(Get) Parnell. I get that Amos had wanted to protect// himself.
**Peyton:**	//He wanted- Yeah, he had his reasons. Maybe it was because Parnell was a big jerk with a big ego. [1] [1] Or maybe because he was trying to protect his sisters.
**Jordan:**	[1] But like… [1]
**Jordan:**	But it doesn’t mean to take the life from [1] someone. [1] That’s a little (mistaken).
**Peyton:**	[1] I know. [1]
**Ryan:**	But like [1] Parnell [1] was drinking and he was trying to hurt- well he did hurt Dovie just because her older sister did not want to marry him.
**Peyton:**	[1] I understand. [1]
**Peyton:**	Beca- Well, here’s what she said, “I wouldn’t want to marry you, even if you were the last man on earth.”
**Jordan:**	And I get that he hurt those and… [1] [1] And when he took the dog, ‘cause he was mad about that; but he didn’t need to get his life taken out of his life. Would you want- If you’d been a terrible snake like him, would you want someone to kill you?

The high-performing group, on the other hand, actively engaged with one another’s ideas and considered their ability to do so both a source of group focus and pride as shown in the debriefing comments. Their growth in social dynamics and intellectual collaboration can also be seen in the following excerpt from week 6. Students in this excerpt collaboratively weighed the bad choices made by Parnell and the other characters’ need for self-defense with the severity of Parnell losing his life.

**Table:** 

**Elliot:**	Yeah, I agree with Parker because… um like, sometimes you need somebody to protect you if you can’t do it.
**Harper:**	And um… Like, if I saw a man did this (stuff) to my sister, I would try to protect my sister. So, uh, yeah. [nods]
**Teacher:**	But he killed someone.
**Jaymie:**	Um, but he killed someone to stood up for her sister because her sister showed, um… His sister showed him how to read, and read lips?
**Parker:**	Well, he killed someone to, basically… It’s not because he didn’t… It might have been be he also didn’t LIKE him, but I mean… He still tried to attack um…
**Jaymie:**	Dovey.
**Parker:**	Dovey, and that’s basically self-defense for Dovey.
**Jaymie:**	(But)//
**Harper:**	//And Parnell had the dog? And Dovey was just trying to get them back, and she couldn’t. So, Amos was probably helping Dovey, and Huck or Tom, whatever dog he had, because he probably loved both of them a lot, and he didn’t want to see neither one of them die, or anything.
**Jaymie:**	I think Parnell kind of deserves it because he was being mean to Amos and Dovey.
**Elliot:**	I// agree with Jaymie.
**Parker:**	//And he… And he like, he basically, like basically, tried to hurt Dovey, and that was wrong.
**Jaymie:**	Even though that Dovey didn’t do anything.
**Harper:**	But also, I agree with (all of these three). I don’t think that he should have lost his life. I don’t think he should have died though.

This group worked together to consider as many ideas as possible, to challenge each other, and to build on each other’s ideas. Because of this, the students in the high-performing group increased their idea building and their contributions over time. This indicates that students were not only voicing more ideas, but were able to relate those ideas to one another to build increasingly complex social arguments. The qualitative evidence also supports the results of transcript and video coding. While the coding results showed that the high-performing group engaged in more equitable social interaction and greater numbers of idea building over time, the qualitative evidence supports that the social interactions may have driven the cognitive changes. In the low-performing group, on the other hand, we see social interactions apparently driving a decrease in cognitive engagement and intellectual collaboration. These findings are also evident in the conflict-dense discussion 4 segments presented in the Appendix.

### The Role of Teacher Scaffolding

As mentioned previously, the high-performing group began with positive social dynamics but lower levels of social reasoning compared to the low-performing group. The teacher was quick to notice that the high-performing group needed encouragement to consider multiple perspectives and engage in higher level thinking. Thus, she started prompting them to consider alternative viewpoints, while also modeling perspective taking and argumentation. In the low-performing group, the teacher seemed pleased with the advanced social reasoning by a few students but realized that this group had other students who were quiet and disengaged. She then began focusing her attention on these disengaged students by encouraging them to speak repeatedly. This, however, led her to neglect the students who participated fully from the beginning. She did not intervene in turn-taking or other social relations and decreased her cognitive scaffolding as she focused more on equal turns of talk. Even when students looked to her for social support, she did not intervene in the social aspects of the discussion outside of simple participation. In this way, we see her decreasing the authority she gives to students in the discussion as she increasingly controls access to the conversational floor.

The high-performing group members were consistently cognizant of the norms of having an open discussion, maintaining mutual respect, and ensuring equitable participation. Therefore, the teacher seemed to put all her attention on scaffolding the students’ intellectual collaboration. She prompted them to provide reasons for their opinions while encouraging them to make connections to their life and to the texts. She demonstrated and modeled perspective taking by explaining the thoughts and feelings of the characters in the story and what she would have done in their position. She treated students’ ideas as equal to her own and took little control over the discussion mechanics, further increasing the already-high relational equity in this group. On the other hand, in the low-performing group the teacher decreased relational equity over the weeks by controlling access to the floor, dominating the power within the group, and not responding to students’ help-seeking. Table 6 shows the teacher’s social scaffolding in both groups. Examples can be seen in the transcript excerpts below, and Table 7 outlines all instances the teacher’s intervention in both groups, excepting interjections and demonstrations of understanding (e.g., “oh, ok”).

**TABLE 6:** Counts of social scaffolding codes.

	Low group			High group		
Week	2	4	6	2	4	6
Relational equity	2	2	5	1	1	7
Participatory equity	1	3	2	0	1	1
Participatory inequity	3	2	0	0	2	1

**TABLE 7:** Teacher’s cognitive scaffolding counts.

	Low group		High group	
Week	2	6	2	6
Open questions	3	4	3	8
Redirection to the big question	3	0	0	1
Modeling reasoning	0	2	3	5
Playing devil’s advocate	0	1	0	5
Presenting hypotheticals	0	0	0	3
Prompting individuals	1	8	0	1
Clarifying questions	1	3	1	1
Low-level support (i.e., vocabulary, giving instructions)	0	4	0	0
Praise	0	3	1	3

Overall, while the teacher’s initial scaffolding in both groups of students was quite similar, by the end she served a drastically different role in each group. In the low-performing group, the teacher served more and more as an authority, often posing a question and having each student respond to it directly. She did not challenge students when ideas did not align with previous comments or encourage them to take one another’s ideas into account in their future considerations. This contributed to the lack of collaboration and relational equity in the low-performing group, as, at times, the teacher actively discouraged collaboration by ignoring student comments to ask a different student to respond to an earlier question she had posed. The following is an example of this from week 6.

**Table:** 

**Teacher:**	‘Cause protecting your family’s worth lying for?
**Jordan:**	[nods] Mhm [affirmative].
**Ryan:**	Um, yeah.
**Teacher:**	You think it is?
**Ryan:**	I think it is.
**Teacher:**	Do you think it is, Peyton? It’s worth lying to protect your family? Yes or no, and why?

In the high-performing group, by contrast, the teacher posed ideas and asked students to consider them without putting her contribution on a higher level than the students’. She built questions and ideas from students’ and seemed genuinely interested in students’ input. In this way, she indirectly encouraged collaboration and built relational equity in the high-performing group. An example is seen in the excerpt from week 6 below.

**Table:** 

**Elliot:**	I agree, also, because they already hated Amos enough, and probably would in jail, they would hated him more.
**All:**	[pause × 7 s]
**Teacher:**	But now, everyone hates the other guy.
**Elliot:**	Wait, the guy who… the guy…
**Harper:**	That got killed.
**Teacher:**	Yeah, he’s being blamed.
**Elliot:**	Ohhhh…
**Teacher:**	How do you choose whose life is more important?

## Discussion

This comparative case study presents how two groups of students who were seemingly similar in their initial social reasoning, academic achievement, and peer acceptance engaged in an intervention called Collaborative Social Reasoning (CSR) and ended up with contrasting levels of social reasoning at posttest. We explored the role of social and cognitive processes and the roles of teacher scaffolding in the dynamic evolution of both the groups. Despite the fact that the two groups of students were facilitated by the same teacher, our findings revealed notable differences between the groups regarding three areas of discussion process: social dynamics, intellectual collaboration, and teacher scaffolding. Specifically, the two groups showed different trajectories of change in relational equity, participatory equity, and idea building. While these practices decreased in the low-performing group, they increased in the high-performing group over time. The ways in which the teacher facilitated the two groups also demonstrated qualitative differences. The teacher seemed to heighten the trends naturally occurring in the students’ social dynamics and intellectual collaboration.

### Social Dynamics

With regard to participatory equity, overall, we observed fewer instances of it in the high-performing group than the low-performing group. In reviewing this group’s discussions, it seems that this is due to the fact that all students were engaged and participating, so invitations to speak were less necessary. When all members of the group elected to share, no one was left sitting silently, and thus, the conversation flowed smoothly and naturally. There was little need for explicit invitations for group members to share, resulting in fewer instances of explicit participatory equity. In contrast, some students in the low-performing group voiced their own opinions to appease the teacher and then returned to silence, without really engaging with ideas or peers, while some other students rarely participated at all. These trends frustrated some students who tried to engage with one another, reducing collaboration over time and leading to greater teacher control.

In terms of relational equity, in the high-performing group students showed respect for the ideas of others, even if it were different from their own. No one seemed to dominate over others. In contrast, in the low performing group, students tended to prioritize their own ideas at the expense of their classmates’ and were not demonstrating respect or value for differences of opinion. Analysis of their expressions and body language further revealed animosity. These students not only generated more conflicts for the floor than the high-performing group over time, but also failed to attend to peer’s speech and adjust their own accordingly. Over a period of time, all of this led to lowered engagement. It was interesting to see how this group, despite starting off on a relatively good note, were not able to balance their social dynamics throughout the intervention. This is in line with Shah and Lewis’s (2019) analysis of social interactions that it takes a series of such small incidences which can eventually accumulate over time and influence group collaboration. In this case, small instances of negative interactions such as interruptions added up over time to worsen the group’s collaboration.

The poor social dynamics of the low-performing group also increased because the teacher began prioritizing the need for silent students to participate over engaging with the ideas of the already-participating students. She began posing a question and asking students to respond to it, instead of considering and building on the ideas that were shared. Chen and Jiang (2004) emphasized the need for teachers to balance multiple dimensions of a discussion while providing contingent scaffolding. The teacher in this study did not maintain such a balance in the low-performing group, probably because of the difficulty in dividing her attention between scaffolding participation and cognitive processes. It is possible that she believed equal numbers of turns of talk was prerequisite for a collaborative discussion and chose her scaffolding strategies accordingly. Future research is needed to identify strategies for finding appropriate balance in cognitive and social scaffolding. In contrast, the high-performing group was able to manage their own social dynamics effectively, and therefore the teacher’s focus on cognitive dimension of the discussion was appropriate to the group’s needs. In the low-performing group, the teacher’s singular focus on cognitive but not social processes was detrimental, and this group spiraled into ineffectiveness over time. These findings thus suggest the reciprocal influence between teacher scaffolding and student group processes.

### Intellectual Collaboration

Our study suggests that intellectual collaboration, including idea building and argumentation, is possible only when positive social dynamics are in place. This is aligned with Vygotsky’s (1934) theory about the intricate relationships between thoughts and affect. In order to build ideas upon others’, group members must be willing to listen to each other’s ideas and to respect different opinions and values. In the low performing group, some of the students focused only on voicing their own opinions, without paying attention to what the others were saying. Some of these students did not appreciate differences in opinion and thereby, did not pick up on each other’s ideas. The high-performing group, on the other hand, actively engaged with one another’s ideas and considered their ability to do so as a source of group focus and pride. They were happy and felt rewarded to have such productive discussions.

The two groups of students’ intellectual collaboration also seem to be affected by how they interacted with the teacher. In watching how the teacher interacted with the students during the discussions, it did seem that high-performing students responded to the teacher’s scaffolding of their intellectual collaboration in a way that enhanced the discussion. When the teacher set up a positive social norm by showing interests in and value for student ideas, the other students in the group followed the norm. Because the teacher engaged in the discussion with the students, her cognitive scaffolding enhanced the opportunities for students to engage in collaborative idea building. The students in the high-performing group therefore were able to actively engage with one another’s ideas and weigh different perspectives by providing detailed explanations. This is aligned with Vygotsky’s (1934) theory that learning occurs through social discourse and collaboration. However, students in the low-performing group became visibly less engaged, with only a few students voicing their opinions in the later discussion. They seldom questioned another’s idea in a constructive way, built upon an idea, or provided a different viewpoint or evidence to support another’s idea. In this group, the teacher acted more as an authoritative figure, choosing students to speak in turn and rarely engaging herself with student ideas. Both the lack of positive social dynamics and the loss of the teacher’s cognitive scaffolding prevented this group from developing the social discourse that Vygotsky (1934) suggested was so critical for learning. These trends could be one of the main factors that explain the difference in both the groups of students’ social reasoning at posttest. These findings also support previous research that has shown how engaging with others’ ideas, providing explanations, considering multiple representations are essential for students’ learning (Warner, 2008; Webb et al., 2014; Ing et al., 2015).

### The Roles of Teacher Scaffolding

**FIGURE 2:**
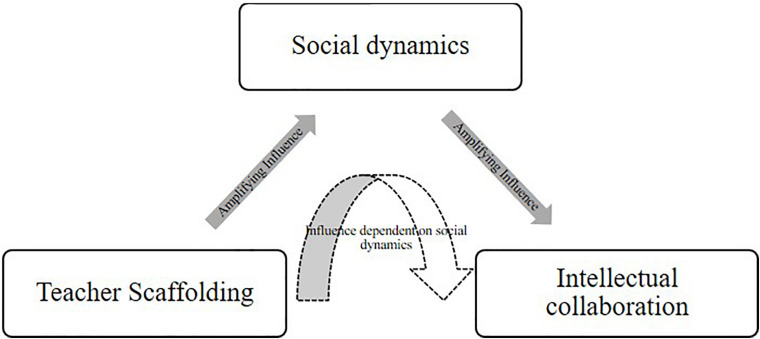
Supported system of small group collaboration.

In a productive collaborative discussion, intellectual collaboration and social dynamics are interrelated (Anderson et al., 2001; Engle et al., 2014), and the teacher serves to support both intellectual collaboration and social dynamics through cognitive and social scaffolding. Interestingly, what we observed in this study was the teacher amplifying existing patterns of relationships between social dynamics and intellectual collaboration in the two groups. This aligns with the bi-directional view of teacher scaffolding, meaning that the teacher both influences and is influenced by the students they are scaffolding (Chen and Jiang, 2004; Webb et al., 2006). In responding to each groups’ existing patterns of interaction, the teacher functioned as a heightening influence on existing patterns. The high-performing group was able to manage their own social dynamics effectively which seemed to facilitate their intellectual collaboration over time. The teacher was able to further this trend by increasing her use of cognitive scaffolding strategies, including open questions, playing devil’s advocate, modeling reasoning, and presenting hypotheticals. These interactions are illustrated in Figure 2. By putting her own ideas into the discussion for consideration, the teacher served to improve argumentation and, indirectly, relational equity. This seemed to give students increasing motivation to value and solicit one another’s opinions, which then further increased positive social dynamics. Even though the teacher’s instances of explicitly referencing relational equity increased in the low-performing group over time, the teacher’s scaffolding does not support these professions; she increases her control of turn-taking and provides low-level support in this group instead of increasing her engagement in equitable discourse. As the focus increasingly became encouraging individuals to talk, there was less cognitive interaction and therefore, less intellectual collaboration in the group.

In considering the implications of this study, it is possible that these findings could inform how collaborative learning is handled. While these findings are preliminary, if future work also finds that the success of intellectual collaboration is dependent on social dynamics, more emphasis would need to be placed on social interaction in preparing and structuring collaborative learning. Teachers would need to be trained specifically in how to balance their scaffolding between social support and cognitive support while still giving students interpretive authority within the work. This could help ensure that the reciprocal influence between social dynamics and intellectual collaboration is beneficial rather than detrimental to the success of the activity.

### Limitations and Future Directions

This research illustrates the ways in which social, intellectual, and instructional factors are inextricably linked in collaborative classroom settings. Too often, these factors are studied separately, which does not enable a comprehensive representation of the complexity of classroom systems. Additionally, these findings point to social dynamics as the driving factor in the groups we studied, which may have implications for how teacher training and collaborative scaffolding take place in the future. These connections must be explored in more detail and in more settings in order to determine whether the patterns identified here are consistent.

Despite the study contributions, there are limitations to this work. As noted, the small number of groups and singular social setting decrease the generalizability of our findings, although the analyses performed in this study are not intended to test any causal relationships. In addition, transcript coding was mainly based on three of the 6 weeks’ discussions due to the labor-intensive process of transcription and coding. The variables that were coded on these 3 weeks of transcripts show a generally linear trend due to the number of time points analyzed. It is possible that the change in these variables is less linear than three timepoints show. Another limitation of the study was that there is limited analysis of post-intervention outcomes. It is not known whether the success of the groups had meaningful implications outside of the discussions, though we did see changes in social reasoning in the high-performing group as noted in the group comparison section. These areas provide fruitful next steps for future research.

This paper provides an initial look at the social, cognitive, and teacher factors within a small group collaborative learning activity as a system, rather than as independent factors, making it unique in its contribution to the field. We found that these factors are inherently interconnected when examining the functioning of the small group, or system, which indicates that work looking at only one of these areas may not accurately represent the learning system. Moving forward, more research should undertake a more holistic research approach so that we can build understanding of the relationships between well-studied individual factors.

## Conclusion

This paper provides an important initial step on this journey and provides evidence that teacher intervention in learning activities may amplify existing patterns rather than build more effective systems. If this trend is found in future work, this will have major implications for teacher training. Finally, this work supports the existence of a critical link between social dynamics and intellectual collaboration and indicates that the connection between the two may be deeper and more intertwined than previous work has suggested. While it is expected that teachers influence power dynamics and equity in the classroom, it is interesting that in this study the teacher heightened the existing social and cognitive relations in the groups. In the group that began with positive social dynamics, she heightened equity and contributed to intellectual collaboration. In the group that began with poorer social dynamics, even with slightly better reasoning, she worsened existing problems by affording less and less power to students, increasing what began as moderate inequity and ended as high levels of inequity.

In considering what these findings mean for collaborative learning more broadly, there is no way to know from these data whether similar findings would be seen outside of these small groups. However, there is an interesting question about interacting factors in collaborative learning that is raised by this work. If social dynamics are, as we found here, the driving force behind the success or failure of collaborative learning, then it is even more critical that students are taught to interact productively in the classroom and that positive relationships are supported. Furthermore, if teachers do indeed serve to amplify existing dynamics in other settings, then research on how teachers can productively intervene to overcome negative social dynamics and support collaboration will be critical.

The consistency in the findings across factors also points both to the validity of the findings and to the interrelatedness of the three factors being studied. While separate examples were provided throughout the findings, a single excerpt represented evidence of multiple findings in several cases. While the directionalities of influence do not follow the ideal hypothesized pathways, the connectedness and relatedness of the factors was as complex as our initial figure predicted. This is further evidence for the need to avoid research that looks at classroom factors in a vacuum and move to work that considers cognitive and social systems more holistically.

## Data Availability Statement

The datasets presented in this article are not readily available because, as the data consist largely of videos and video transcriptions, they necessarily contain considerable identifying information. As such, limited access to the data corpus can be provided to individuals not approved by the IRB. Additional examples, coding results, and limited identified data can be provided by contacting the first or third authors. Requests to access the datasets should be directed to kraatz.3@osu.edu; lin.1653@osu.edu.

## Ethics Statement

The studies involving human participants were reviewed and approved by The Ohio State University Internal Review Board. Written informed consent to participate in this study was provided by the participants’ legal guardian/next of kin. Written informed consent was obtained from the individual(s), and minor(s)’ legal guardian/next of kin, for the publication of any potentially identifiable images or data included in this article.

## Author Contributions

EK was responsible for the majority of the planning, data analysis, and writing of this manuscript. MN contributed significantly to the planning, analysis, and writing of the manuscript. T-JL oversaw the project, provided advice and revisions, and was the primary investigator on the larger project from which this data was taken. M-YH, SH, and SK were part of the research team on the larger project and contributed through intellectual collaboration, coding and theoretical assistance, and manuscript revisions. SS contributed to the planning and implementation of the data collection and is now deceased. All authors contributed to the article and approved the submitted version.

## Conflict of Interest

The authors declare that the research was conducted in the absence of any commercial or financial relationships that could be construed as a potential conflict of interest.

## Appendix

**TABLE A1:** Discussion four’s most conflict-dense excerpts by group.

Low-performing group		High-performing group	
Peyton	Hold on. I would drive it all over the neighborhood and show ’em, but I wouldn’t drive it toward the South and show that tailgate thing.	Harper	I agree with Elliot, because like when the cop pulled him over and put him in the handcuffs and stuff because it just would be unusual for a black man at that point of time to be driving a gold Cadillac, a Cadillac though. That is all gold and that just to be driving a Cadillac. [1] [1] And then he’s a black man.
Jordan	I wouldn’t really…//	Jaimie	[1] I’m not… [1]
Teacher	//Why? Why not South?	Spencer	I’m not sure if they had license plates back then? I don’t know. But if they did, they could have read his license plate and said it was from Ohio and since he lived, and since the family lived in Toledo, they would probably know that “Oh that’s a city that doesn’t do segregation so we could we could just let him go because he probably did just buy the car he didn’t steal it.”
Jordan	I…//	Elliot	I disagree with Jaimie because what if the cop thought differently and didn’t give the father a chance to talk?
Peyton	//Well, because it’s dangerous!	Spencer	Well they can’t… well yeah. True.
Jordan	I would actually just, if I had a Lamborghini, I wouldn’t go out there just bragging that I had a Lamborghini. I would just use it to get to somewhere because not everyone has the same thing as we do.	Harper	Um I agree with Elliot because like if the white man was racist he probably just would have put the black man in jail anyway. Probably because he didn’t like black people.
Ryan	And the family, they could have went during nighttime, where people weren’t really out- or their family could’ve just came to them instead of them having to go to the family.	Cameron	I switch my decision to say that they should be able to take it down because of what Jaimie said before that it was after the rights were making… that after the rights were made and the now they can’t do slavery and that they could take the car wherever they wanted if they paid good money for it.
Teacher	That’s a good point. So you’re saying the family could have drove to them instead of them risking them going dangerous down South. Is that a lot to ask of the family?	Jaimie	And…//
Jordan	No.	Elliot	//I disagree with Cameron because what if the cop don’t know that you bought and they probably thought that you stole it?
Peyton	No.	Spencer	Well normally when you buy a car- well normally when you buy a car you like there’s things in the car [1] that states that it’s [1] yours. Yeah a title that states that you have… that it’s your car so they couldn’t just like, like think that he stole the car. They have to actually find evidence why they think they stole the car.
Ryan	No because they chose to live there.	Harper	[1] Title. [1]
Jordan	Yeah.	Harper	What if…// [to Jaymie] Go ahead.
Teacher	It was their choice. Yeah, because didn’t the dad used to live there? And then they moved up to Ohio before?	Jaymie	//I… Alright. I changed my mind because if they knew that they couldn’t drive expensive cars, like, they will get in more problems.
Peyton	Well, the car. If the car was a pet and it drove all the way to the South with me in it, I would say, Bad car, bad!// And…	Spencer	But he wants to show off like what he’s doing and wants to show what he’s been doing working and so I think that’s a good idea to show them that he’s proud of what he does and…//
Jordan	//The mom also got angry about something.	Harper	//Um I disagree because like that’s like a reason why I wouldn’t drive that down south because like when you try to show things off, people mostly want it and they’ll try to take it. That’s why I don’t show things off. Because if you want to show a car off and you park that car, somebody wanna come up and try to steal it if you’re showing it off.
Teacher	Why do you think she got so angry?	Teacher	Do you have personal experience with that?// Or just seen people do that?
Jordan	Because they were going to drive up South?	Harper	//Um.. yes. Like I said I was showing my stuff off before and my friend stole it and I wasn’t going to do that after that.
Ryan	They were gonna buy a home with the money and save up to have a better neighborhood to live in. [1] [1] Less chances of being robbed or killed or…	Cameron	Oh what did he steal?
Jordan	[1] So that they wouldn’t… [1]	Harper	He stole my toys. [All laugh] He stole my toy out of my toy bin.
Jordan	Or uh… arrested at the same time.	Teacher	I’m so sorry to hear that, bud.
Ryan	Maybe a bit safer.	Spencer	Um well I think that if he’s like showing- if he’s like telling… Well he’s-if he’s showing them that he has the gold Cadillac I think it’s a good idea because he’s showing them that just not, it doesn’t mean if you’re colored you can’t get the respect that you want. Well if you’re white you don’t always have- if you’re white you don’t have to always put people down because of their color and he’s like probably telling them like “I have respect for other people and I’m going to show- and I want people to have respect for me.”s
